# Recommendations for reporting and evaluating proton therapy beyond dose and constant relative biological effectiveness

**DOI:** 10.1016/j.phro.2024.100692

**Published:** 2024-12-25

**Authors:** Armin Lühr, Dirk Wagenaar, Daniëlle B.P. Eekers, Lars Glimelius, Steven J.M. Habraken, Semi Harrabi, Miranda C.A. Kramer, Ranald I. Mackay, Ana Vaniqui, Alexandru Dasu, Damien C. Weber

**Affiliations:** a Department of Physics, TU Dortmund University, Dortmund, Germany; b Department of Radiation Oncology, University Medical Center Groningen, University of Groningen, Groningen, the Netherlands; c Department of Radiation Oncology (Maastro), GROW School for Oncology and Reproduction, Maastricht University Medical Centre+, Maastricht, the Netherlands; d RaySearch Laboratories AB, Stockholm, Sweden; e HollandPTC, Delft, the Netherlands; f Department of Radiation Oncology, Leiden University Medical Center, Leiden, the Netherlands; g Heidelberg Ion Beam Therapy Center (HIT), Department of Radiation Oncology, Heidelberg University Hospital, Germany; h Division of Cancer Sciences, School of Medical Sciences, Faculty of Biology, Medicine and Health, The University of Manchester, Manchester, United Kingdom; i Christie Medical Physics and Engineering, The Christie NHS Foundation Trust, Manchester, United Kingdom; j Belgian Nuclear Research Center (SCK CEN), Mol, Belgium; k The Skandion Clinic, Uppsala, Sweden; l Department of Immunology, Genetics and Pathology, Uppsala University, Uppsala, Sweden; m Center for Proton Therapy, Paul Scherrer Institute, Villigen PSI, Switzerland

**Keywords:** Proton therapy, Relative Biological Effectiveness, Linear Energy Transfer, Radiation-induced toxicity, Educational needs, European Particle Therapy Network, EPTN

## Abstract

**Background and purpose:**

In proton therapy, a relative biological effectiveness (RBE) of 1.1 is used to convert proton dose into an equivalent photon dose. However, RBE varies with tissue type, fraction dose, and beam quality parameters beyond dose such as linear energy transfer (LET) raising concerns about increased local effectiveness and potential toxicity. This work aims to harmonize quantities used for clinical consideration of variable RBE for proton therapy.

**Materials and methods:**

A survey was distributed to proton centres to determine agreement on RBE-related concerns and clinical implementations. A subsequent clinical expert meeting facilitated by the European Particle Therapy Network was held to achieve consensus and to make clinical recommendations how to prescribe and report beyond using dose and constant RBE.

**Results:**

The survey was answered by 17 out of 23 centres contacted (74%). For proton RBE, most concerns existed regarding toxicity in serial organs, while the assumption of an RBE of 1.1 was considered valid for targets. Most physicists intended to consider a physical quantity beyond dose in clinical decision making.

**Conclusions:**

A constant RBE of 1.1 was the consensus for prescribing dose. However, current practice of recording and reporting dose in proton therapy must be complemented: the recommended quantity beyond dose was the dose-averaged LET in water from primary and secondary protons, normalized to unit density. This will facilitate analyses of treatment data on effectiveness beyond dose and between centres. No consensus on a single variable RBE model was found. More clinical training on proton RBE is needed.

## Highlights

• Dose-averaged linear energy transfer (LET_d_) is to be reported for each patient.
• LET_d_ is to be calculated in water considering all protons but no heavier fragments.
• Toxicity is the main concern with variable relative biological effectiveness.
• No consensus on a single variable relative biological effectiveness model reached.
• More education on LET and radiobiology is needed for proton therapy.

## 1 Introduction

In proton therapy, the application of a relative biological effectiveness (RBE) factor is essential for converting proton dose into an equivalent photon dose. Conventionally, an RBE of 1.1 is employed in proton therapy [1], [2], [3]. Thus, in routine practice, the absorbed dose prescribed using protons is 10 % lower than for an equivalent treatment with photons. A nuanced understanding of RBE is imperative for optimal treatment plans, carefully balancing the goals of tumour control with the preservation of normal tissues.

A high ionisation density has long been associated with an increased radiobiological effect in particle therapy [1], [4]. Several quantities have been proposed to quantify this aspect [5], [6], [7], including microscopic and macroscopic quantities such as lineal energy and linear energy transfer (LET). Correlations between these quantities and experimental radiobiological [8], [9], [10], [11] and clinical results [12] have been sought with varying degrees of success. So far, no single metric for ionisation density has been demonstrated to perform significantly better than others in predicting the modulation of radiobiological effects in clinical cases. Proposed quantities, such as LET, can be precisely determined for monoenergetic particles in homogeneous and well-defined materials. However, calculations for mixtures of materials and for multi-energetic mixed radiation fields, as they occur in clinical practice, require further assumptions that impact the calculated values [13]. For example, Hahn et al. [14] have shown for a multicentric clinical setting that LET calculations and reporting are affected by the weighting used for averaging in multi-energetic fields, the choice of secondary particles considered as well as the physics models and parameters employed in Monte Carlo simulations. Therefore, beyond the standardised dose [1], a quantity needs to be specified in a harmonised way [3], [15], [16] to achieve uniform data recording and reporting in proton therapy [17]. Here, a pragmatic approach might be considered by defining a quantity beyond dose that can be calculated consistently by most clinical centres [3], [14]. It is imperative to reach consensus, as this is a prerequisite for comparing and combining future studies. This in turn will enable the accurate determination of the biological effectiveness of protons to eliminate clinical uncertainty regarding RBE as quickly as possible.

The current use of a single value of 1.1 as RBE was chosen to enable target prescription dose conversion to equivalent photon therapy dose to the target [1]. The use of a constant RBE model is being reconsidered, especially for normal tissues. Several RBE models have been proposed based on cell survival data [10], [18], [19]. However, pathways leading to normal tissue damage are different, and not all pathways are equally influenced by factors such as LET [20]. Nevertheless, the effect of LET on the incidence of radiation induced contrast enhancement in the brain has been shown by several independent centres [21], [22]. Conversely, some centres reported less clear or even no correlations [12], which may be due to small patient cohorts or little consistency in some of these studies.

There are four general types of RBE models, with the current constant RBE model being the simplest. Linear RBE models are a first-order approximation of RBE, simply increasing RBE linearly with LET. Such models have been developed as a tool for a simple risk assessment regarding variable RBE in treatment planning [23], [24] or to fit clinical data [21], [22]. Phenomenological models also make no attempt to incorporate underlying biology, but try to fit a function, which is based on the linear-quadratic radiation response model, on experimental data for cell survival [10], [19]. These models typically incorporate the α/β value, fraction dose and LET [10], [19]. Mechanistic models predict RBE by modelling the mechanism for damage and radiation response and may ideally be applicable to both proton and ion therapy [25], [26].

An essential next step to address challenges associated with RBE is to align the experts’ knowledge and the practical concerns of clinicians working in proton therapy. The primary goal of this work was to achieve a consensus and formulate recommendations regarding the reporting beyond dose using LET and RBE parameters of treatment plans for patient cases, including information relevant for research purposes.

## 2 Material and methods

### 2.1 Survey

The urgent need for harmonising the use of quantities for clinical consideration of a variable RBE was recognized by several international radiotherapy-related organisations including the European Particle Therapy Network (EPTN), the American Association of Physicists in Medicine [17] and a collaboration of Nordic centres in Europe [27]. Using the framework of EPTN, a task force of the European Society for Radiotherapy and Oncology (ESTRO), a two-step approach was employed to reach consensus and formulate recommendations among practitioners and clinical researchers consisting, first, of a survey among proton therapy centres and, second, an expert meeting by radiation oncologists and clinical physicists.

The survey was set up as an online questionnaire using SurveyMonkey (SurveyMonkey, San Mateo, USA). The questionnaire was designed to be filled in twice by each institute, once by a radiation oncologist and once by a clinical medical physicist. A total of 38 questions were included, consisting of 8 general questions, 11 questions for radiation oncologists and 19 questions for clinical medical physicists. The survey was sent out to all European proton therapy centres but excluding centres with only a fixed beamline used for eye treatments. Some centres filled in the questionnaire multiple times as a radiation oncologist or physicist. These entries were resolved by combining similar answers and changing conflicting answers to “do not know”.

### 2.2 Clinical expert meeting

A dedicated one-day meeting was held in October 2023. The meeting was attended by clinical medical physicists and treating physicians from most (19 of 23) European proton therapy centres and delegates from five institutions including national standard labs, cancer research centres and medical software industry. The objective was to identify consensus regarding the reporting of radiation data within a clinical context that extends beyond dose using LET and RBE parameters, with a particular focus on patient cases. Proposals for statements and recommendations, whether derived from the survey findings or from suggestions made by clinical participants, were discussed in terms of the level of consensus, the relevant literature and clinical evidence, if available. The aspects discussed at the meeting are represented in the discussion section of this manuscript.

## 3 Results

**Fig. 1:**
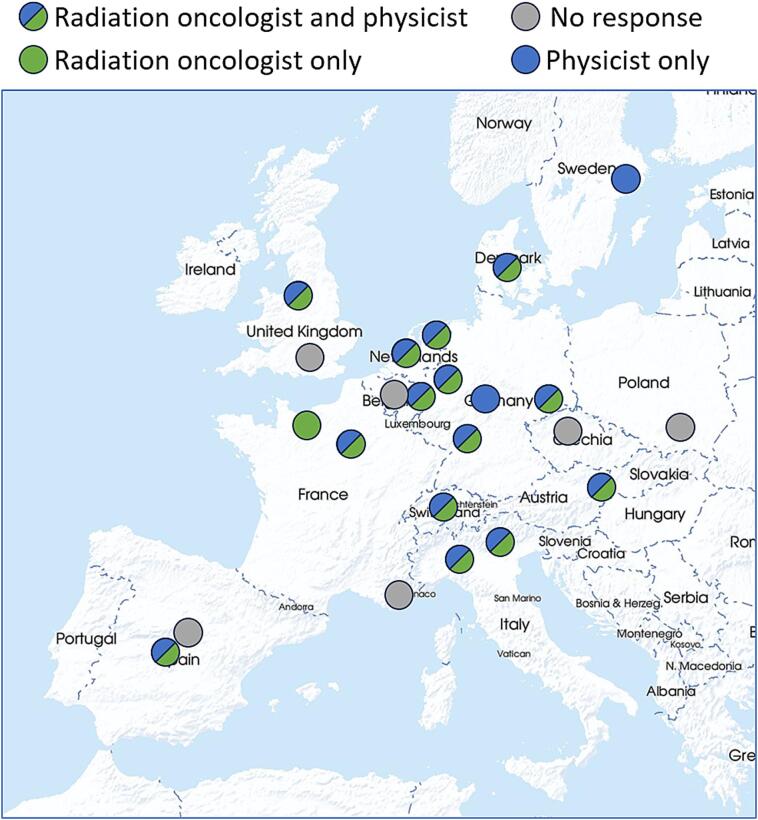
Location of all approached proton therapy centres with indication of their response to the survey. (Original map provided by mapswire.com).

The survey response was 17 out of 23 approached centres (74 %) with a total of 15 radiation oncologists (65 %) and 16 physicists (70 %) responding (Fig. 1). A complete list of questionnaire questions and their composite results are available in the supplementary materials.

**Fig. 2:**
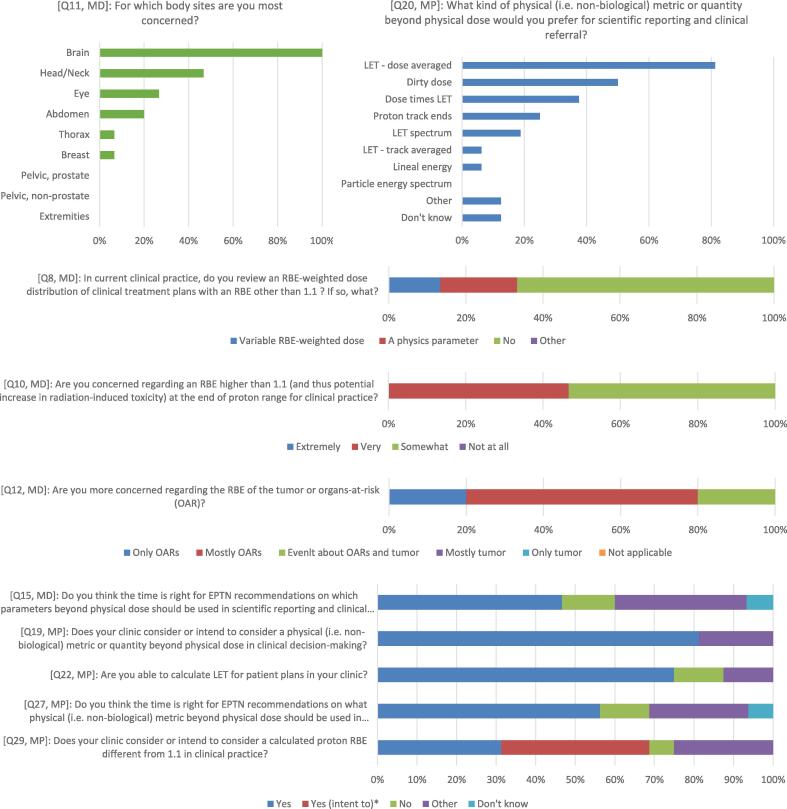
Histograms of the answers to survey questions (Q). Responses from radiation oncologists and medical physicist are labelled MD and MP, respectively. *: Question 29 had the response option “Yes, we intend to consider it”. Such a response option (expressing intent) was not available for the other questions in the panel (15, 19, 22 and 27). EPTN: European Particle Therapy Network, LET: Linear Energy Transfer, RBE: Relative Biological Effectiveness.

Radiation oncologists indicated that they were either somewhat concerned (53 % of 15 replying physicians) or extremely concerned (47 %) regarding an RBE higher than 1.1 at the end of proton range for clinical practice (Question 10 [Q10], Fig. 2). All physicians were concerned with RBE in the body site brain (100 %) and about half for head and neck (47 %), while less were concerned for eye (27 %), abdomen (20 %), thorax (7 %) and breast (7 %) [Q11]. Physicians also indicated they were mostly (60 %) or only (20 %) concerned about the RBE of organs-at-risk (OAR), while only some (20 %) were evenly concerned about the RBE of OAR and tumour and no one only regarding tumour [Q12].

Most clinical medical physicists indicated that they consider or intend to consider a physical (i.e. non-biological) metric or quantity beyond dose in clinical decision making (88 % of 16 replying physicists) [Q19] (Fig. 2). The metric preferred by most physicists (81 %) for scientific reporting and clinical referral was dose-averaged LET (LET_d_), with some (56 %) also indicating dose deposited by protons above a certain LET threshold, a concept also known as “dirty dose” [Q20].

Most radiation oncologists (67 %) replied that their centre currently does not review an RBE-weighted dose distribution of clinical treatment plans using an RBE model other than 1.1 [Q8] (Fig. 2). However, most clinical medical physicists (69 %) indicated that they either consider (31 %) or intend to consider (38 %) a calculated RBE different than 1.1 in clinical practice [Q29]. Most physicists reported that their clinic is able to calculate variable RBE distributions (75 %) [Q22], but routinely this is only done at one clinic [Q33].

Regarding the need for guidelines, about half of the radiation oncologists (47 %) indicated the time is right for recommendations on which parameters beyond physical dose should be used in scientific reporting and clinical referral [Q15] (Fig. 2). Most of the remaining respondents indicated that they would welcome guidelines for scientific reporting but see difficulties for recommendations for clinical referral without further clinical evidence. Similarly, over half of the clinical medical physicists (56 %) indicated they think the time is right for recommendations on what physical metrics should be used in scientific reporting and clinical referral [Q27].

Both physicians and physicists agreed on the need for more education regarding parameters beyond physical dose (93 % of physicians [Q17] and 75 % of physicists [Q37]) and the need for a multi-institutional database for patient outcomes focusing on the question of proton RBE (67 % [Q18] and 81 % [Q38]).

## 4 Discussion

**Fig. 3:**
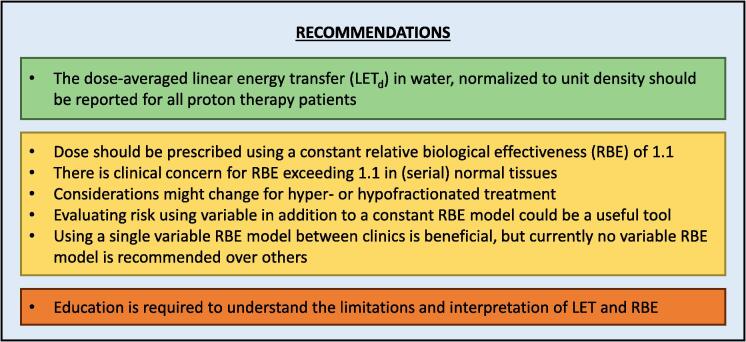
Clinical recommendations on how to move beyond dose in proton therapy to consider variable relative biological effectiveness (RBE): top row (green) indicates physics, middle (yellow) clinical and bottom (orange) overarching aspects. LET: Linear Energy Transfer.

The practical concerns of clinicians working in proton therapy regarding RBE need to be addressed. A consensus was reached on how to move beyond dose based on a survey of proton therapy centres and an expert meeting (Fig. 3). The resulting clinical recommendations are presented below.

It is recommended that the dose-averaged LET in water, normalized to unit density, is computed and reported for all proton therapy patients. Dose averaging should include LET contributions from primary and secondary protons, but not from heavier fragments. And the contribution per particle should be given by the unrestricted LET in water.

The reporting of the LET_d_ in water mirrors the current practice of both prescribing and calculating the dose to water in tissue [1] and will avoid additional sources of uncertainty stemming from the definition of tissue equivalents or their composition [13]. Calculation of unrestricted LET_d_ of only primary and secondary protons improves the precision of the calculation [28] while the cross-sections for heavier particles induce more uncertainty [29], [30]. When possible, the LET_d_ should be obtained and reported as a three-dimensional distribution per plan as well as per beam [31]. Note, however, that the dose-average of the beam LET_d_ distributions does not exactly match the plan LET_d_ according to the definition above, since the absorbed dose includes the contributions from heavier particles.

The LET_d_ has been recommended, since it is the most prevalent form of LET in proton studies [12], [13], [32]. However, it is essential to acknowledge that any single LET figure is only an incomplete approximation of the full LET spectrum. The proposed standardised dose-averaged LET reduces the diversity in the type of averaging, the particles included, the medium and density that LET has been calculated in, and the lower energy cut-off, which are parameters for LET. Nevertheless, further investigation beyond LET_d_ is required to elucidate the relationship between proton RBE and other physical aspects (e.g. microdosimetric quantities, dose rate for temporal aspects, spectrum of particles).

The LET_d_ according to this definition should be computed and reported in addition to all other quantities relevant to the purpose of the investigation. This will provide a common baseline facilitating future result comparisons between centres or *meta*-analyses as well as verifying calculations of different treatment planning systems [17], [31]. For this purpose, it would be desirable if the LET_d_ and its calculation parameters are included in the DICOM-RT standard (cf. [Q26]), which has recently been explored [31].

The existence of multiple Monte Carlo codes advocates for establishing a gold standard against which the performance of individual codes can be measured [31]. In addition, regular benchmarking or audit activities would have to be defined to compare LET calculations among centres for relevant example cases [32], which parallel the dosimetric beam output audits [31]. In this scenario, experimental (micro-) dosimetry could serve as a valuable quality assurance tool for assessing the calculated quantities [31], [33], [34].

The presented recommendations also build upon a previous EPTN report [16], which considers maintaining comparability of clinical dose prescription and outcome data between proton therapy centres of highest priority and consensus on standardising LET calculation to be of paramount importance. In a previous survey, proton therapy centres considered the ability to calculate (any kind of) LET in their clinical setting as the next crucial step in overcoming the RBE issue [3], while the current survey results showed that this is now feasible for most European centres. A European multicentre treatment planning comparison study demonstrated that using the harmonised LET_d_ definition together with one variable RBE model resulted in only minor differences in the RBE-weighted dose between centres, with these differences mainly due to differences in the planned absorbed dose [14], [35]. At the same time, delegates agreed that the search for relevant metrics for ionisation density to correlate with biological effects in proton therapy needs to continue.

Importantly, the regular use of the proposed quantity LET_d_ needs to be supported by educational activities targeted at how it should be interpreted. It is necessary to understand that the LET_d_ will be uncertain in regions where the dose is uncertain (e.g. low-dose regions). Additionally, users should understand that LET_d_ is a very different quantity than dose. Even though high LET_d_ may imply an elevated biological effect [9], in regions with low dose this may not be clinically relevant. As a result, an analysis based solely on the LET_d_ or LET-volume-histograms (LVH) of regions-of-interest (ROI) may lead to misleading results, particularly, for ROI with a heterogeneous dose [36], [37]. Instead, the evaluation of LET_d_ should always be combined with dose before condensing a 3D distribution. Evaluating the product of RBE and dose would prevent these issues, but other solutions have been suggested (e.g. dose-LET-volume histogram [36], “dirty dose” [38], [39]).

Regarding the use of RBE for patient treatment, it is recommended to use a constant RBE of 1.1 for prescribing proton therapy in accordance with ICRU recommendations [1]. This approach is currently preferred even though it is known that RBE varies for both target and normal tissue. It allows clear communication of the absorbed dose delivered to patients and comparison of dose and prescription over time and across proton centres. The discussion at the workshop was limited to conventional fractionation schemes with a fraction dose around 2 Gy (RBE) as these are most prevalent in clinical operation. However, it should be noted that many of the considerations on variable RBE may change when applying hyper- or hypo-fractionated treatment schemes [20], [40].

There is concern that the RBE might be locally higher than the assumed 1.1 across all tissue types and proton dose distributions [16], [20], [31]. In line with the survey outcome, this concern was mostly expressed for toxicity and in particular for body sites close to serially organised tissue such as brain and head and neck and less for abdomen and extremities. There was consensus that the assumption of a constant RBE of 1.1 is conservative, effective and useful for tumour control [17]. Using an alternative variable RBE model as an additional evaluation only for normal tissues for which the RBE is a concern is thought to be a useful tool [31]; either for changing the RBE-weighted dose only for these normal tissues or for identifying areas where elevated RBE might increase the risk for toxicity. Currently, the most common clinically implemented strategy to mitigate the risk of adverse effects associated with variable RBE is to avoid proton beams stopping in OAR [3], [41].

The predicted RBE varies considerably when biological parameters vary, since the complex biological dependencies are difficult to parameterise. In contrast, for most proton RBE models, changes in the physical properties of the beam affect the predictions in a similar way [19]. Each model type has unique benefits. Mechanistical models allow proton RBE calculation to be similar to heavy ion RBE calculation, but their calculation and determination of biological parameters is more complicated [26]. On the other end, linear RBE models are considered elegant due to their simplicity and can be regarded as an approximation of RBE trends but interpreting the exact meaning of the resulting dose is difficult [21], [22], [23]. Phenomenological RBE models offer a compromise as they allow the inclusion of a few relevant parameters while still being relatively straightforward to calculate [10], [18], [19]. Given the uncertainty, models for potential clinical use should be chosen based on their simplicity and ideally ion-independent unless certain features of the models are key to the application or study.

Although no model can be recommended as superior at this time, proton centres can use an RBE model as an instrument to evaluate areas where end-of-range effects may play a role [31]. Consequently, further optimisation may be performed to adjust the spot delivery pattern where the combination of dose and RBE is detrimental to the optimised treatment plan [28], [42], [43]. If this is done, the model, its parameters, and the variable RBE-weighted dose and RBE of 1.1 weighted dose should be recorded. Selecting a single variable RBE model to be used in addition to RBE 1.1 for reporting would facilitate straightforward and effective communication between proton therapy centres [35], [44], while different models can hamper comparison of treatment approaches across centres as observed in carbon ion clinics [25].

In conclusion, the current practice of reporting absorbed dose in proton therapy should be complemented by the recording and reporting of an additional quantity accounting for the increased effectiveness of protons. The LET_d_ in water from primary and secondary protons, normalized to unit density is the recommended quantity beyond dose to be computed and reported for proton plans. For proton RBE, most concerns exist with respect to toxicity in serial organs, while for effects in the targets the assumption of an RBE of 1.1 is considered valid for conventional fractionation. Variable RBE models could be explored as additional instruments to evaluate possible effects arising from increased ionisation density in some tissues/areas. Educational activities on the implications and limitations of the proposed LET quantity and RBE models should be established for clinical personnel in proton therapy centres. Finally, the recommendations should be seen as a step towards reporting biologically effective dose to be followed by others, as there is still a significant lack of knowledge about the radiobiological effectiveness of protons.

## CRediT authorship contribution statement

**Armin Lühr:** Conceptualization, Methodology, Data curation, Writing – original draft, Writing – review & editing. **Dirk Wagenaar:** Conceptualization, Methodology, Visualization, Writing – original draft, Writing – review & editing. **Daniëlle B.P. Eekers:** Writing – original draft, Writing – review & editing. **Lars Glimelius:** Writing – original draft, Writing – review & editing. **Steven J.M. Habraken:** Writing – original draft, Writing – review & editing. **Semi Harrabi:** Writing – original draft, Writing – review & editing. **Miranda C.A. Kramer:** Writing – original draft, Writing – review & editing. **Ranald I. Mackay:** Writing – original draft, Writing – review & editing. **Ana Vaniqui:** Writing – original draft, Writing – review & editing. **Alexandru Dasu:** Writing – original draft, Writing – review & editing. **Damien C. Weber:** Conceptualization, Writing – original draft, Writing – review & editing.

## Declaration of competing interest

The authors declare the following financial interests/personal relationships which may be considered as potential competing interests: LG reports to be employed by RaySearch Laboratories.
