# Supplementary material for: Recommendations for reporting and evaluating proton therapy beyond dose and constant relative biological effectiveness
Source: Phys Imaging Radiat Oncol. 2024 Dec 25;33:100692. doi: 10.1016/j.phro.2024.100692 (PMC11750264; doi:10.1016/j.phro.2024.100692)

Consensus on reporting beyond dose: LET and RBE

Survey introduction

Currently, the clinical standard is the use of a constant relative biological effectiveness (RBE) of 1.1 to calculate the clinical dose distribution. This clinical dose distribution is, therefore, derived only from the physical dose distribution. Some methodologies have been developed to use in addition other quantities beyond dose such as LET distributions and RBE-weighted dose (DRBE) distributions. These quantities are different in that they take the variability of the 'biological' response into account. While potentially helpful, the multitude of these quantities may result in different and inconsistent descriptions of irradiation beyond dose. Therefore, one might consider harmonizing the use of these quantities to facilitate inter-center communication. This could make it easier to guarantee clinical evidence generated in one center to be used in other centers or to pool clinical data in future meta-analyses.

In the framework of ESTRO's European Particle Therapy Network (EPTN), a joint meeting is being prepared by work package 1 (Clinical), work package 5 (Treatment planning systems) and work package 6 (Radiobiology). The aim of this one-day meeting is to investigate whether we can reach consensus on the reporting of LET and RBE parameters of clinical cases and for research. The meeting is scheduled on Thursday October 26th in Manchester (prior to the general EPTN meeting). Additional information will be available soon on EPTN's website.

With this questionnaire, we would like to identify, first, the current use of parameters beyond dose (e.g., LET/RBE) in clinical practice in Europe and, second, areas where we are likely to reach consensus with a questionnaire. In addition, the results will be made publicly available. The questionnaire can be paused and resumed at a later time.

We ask that the questionnaire (estimated duration 10 minutes) is completed twice by your proton therapy facility: once by a radiation oncologist and once by a physicist familiar with the subject. We kindly ask you to complete the questionnaire by September 1st, 2023.

Survey outcome

Section 1. General

[Q1]: What is your full name?

[Q2]: What is your email address?

[Q3]: What is the name of your institution?

[Q4]: In which city is your institution located?

[Q5]: In which country is your institution located?

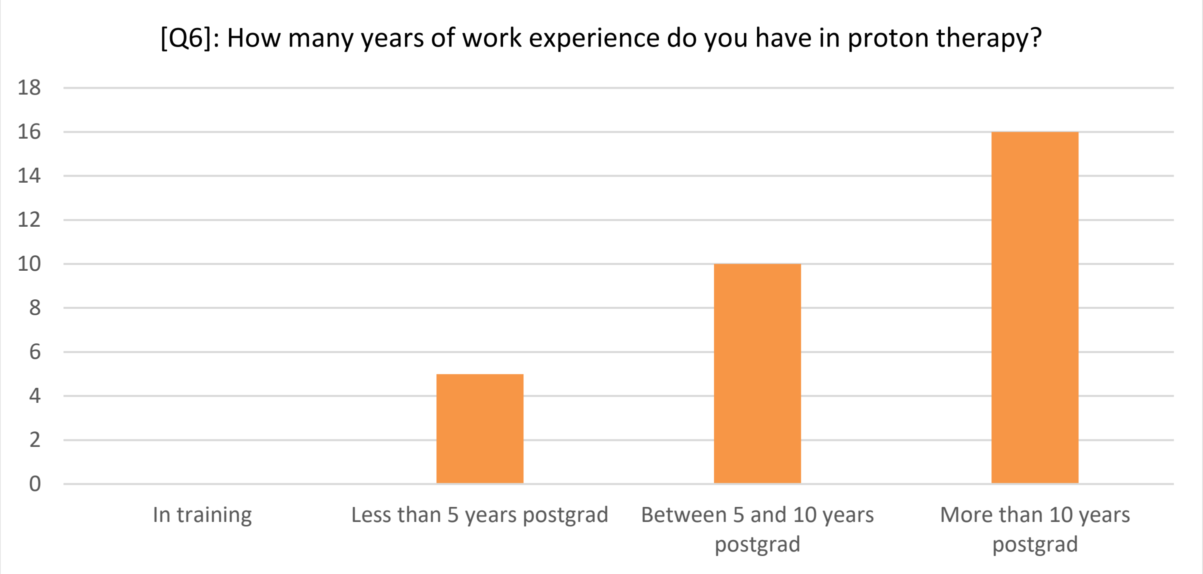

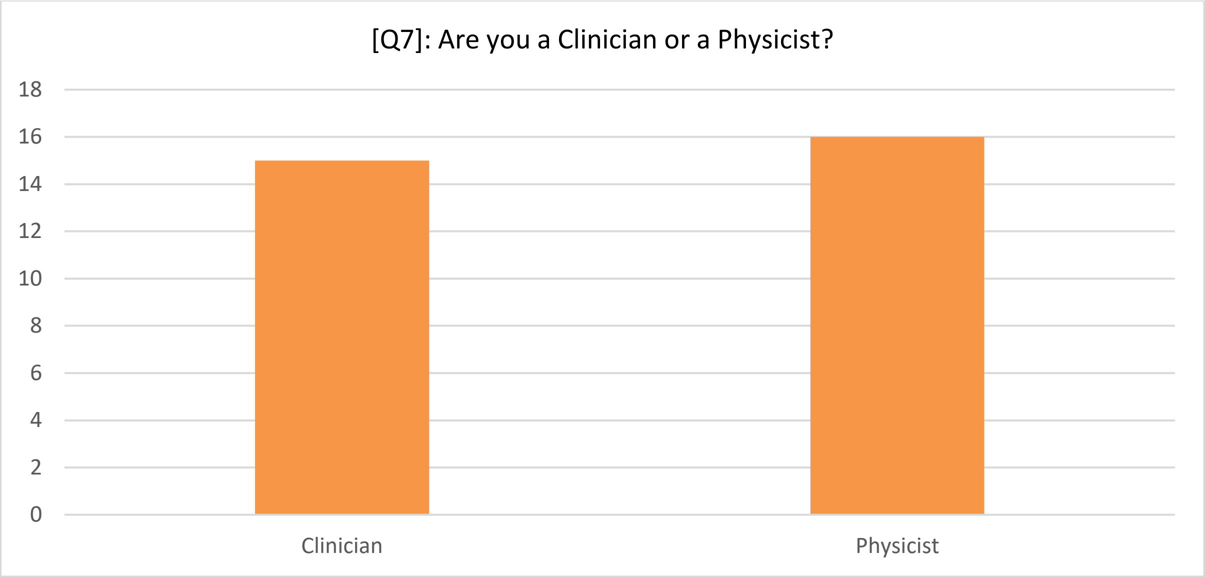
[Q7]: Are you a Clinician or a Physicist?
18
16
14
12
10
8
6
4
2
0
Clinician
Physicist

Section 2: Clinician

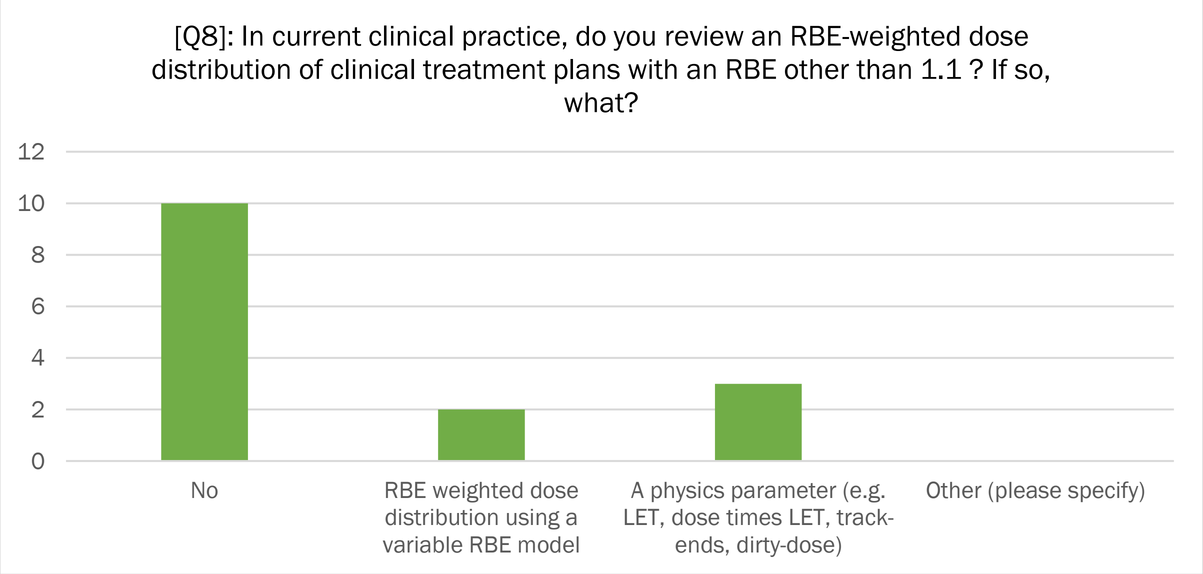

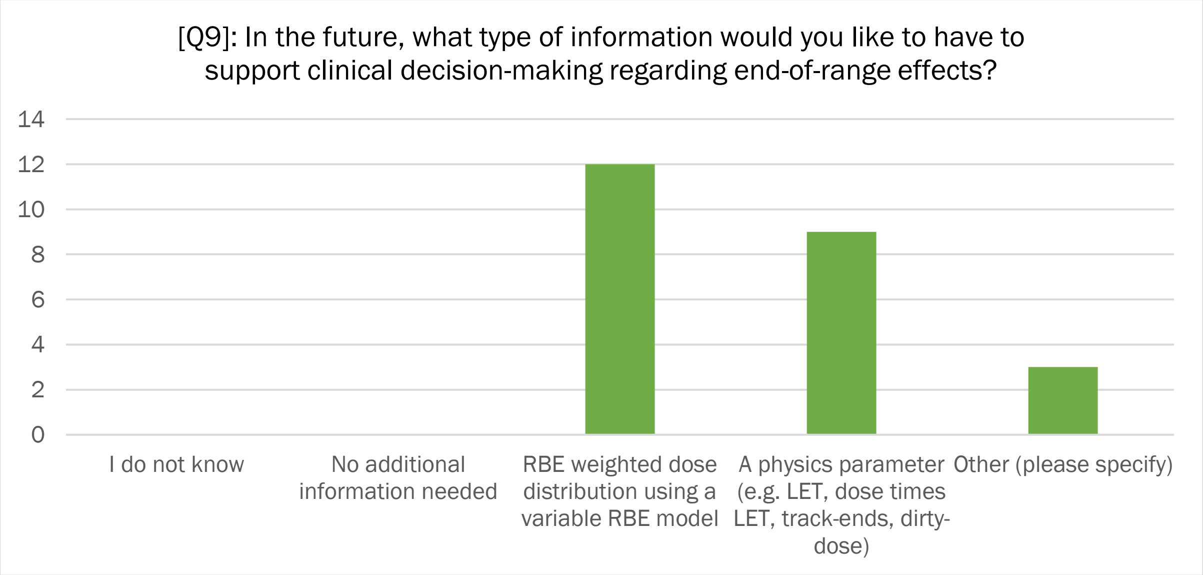

Specification of “Other (please specify)” answers:

- More information on how LET and RBE relate to clinical endpoints
- in addition to LET I would like to routinely get the risk map with estimated POLO (probability of lesion origin)
- A Physics parameter (e.g. LET, dose times LET, track-ends, dirty-dose)

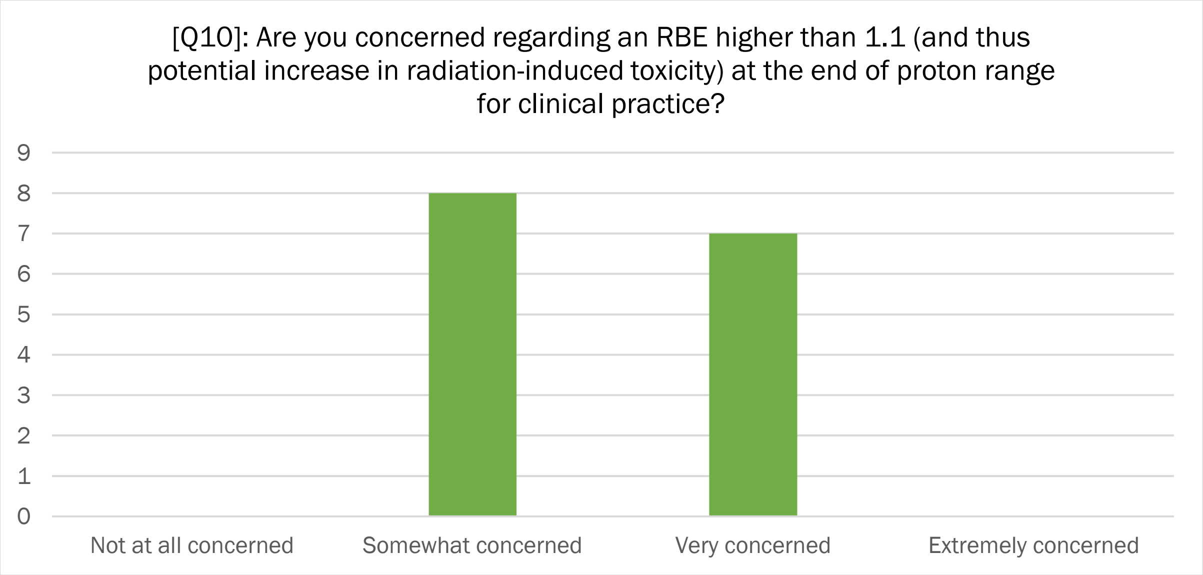
[Q10]: Are you concerned regarding an RBE higher than 1.1 (and thus potential increase in radiation-induced toxicity) at the end of proton range for clinical practice?
0
1
2
3
4
5
6
7
8
9
Not at all concerned
Somewhat concerned
Very concerned
Extremely concerned

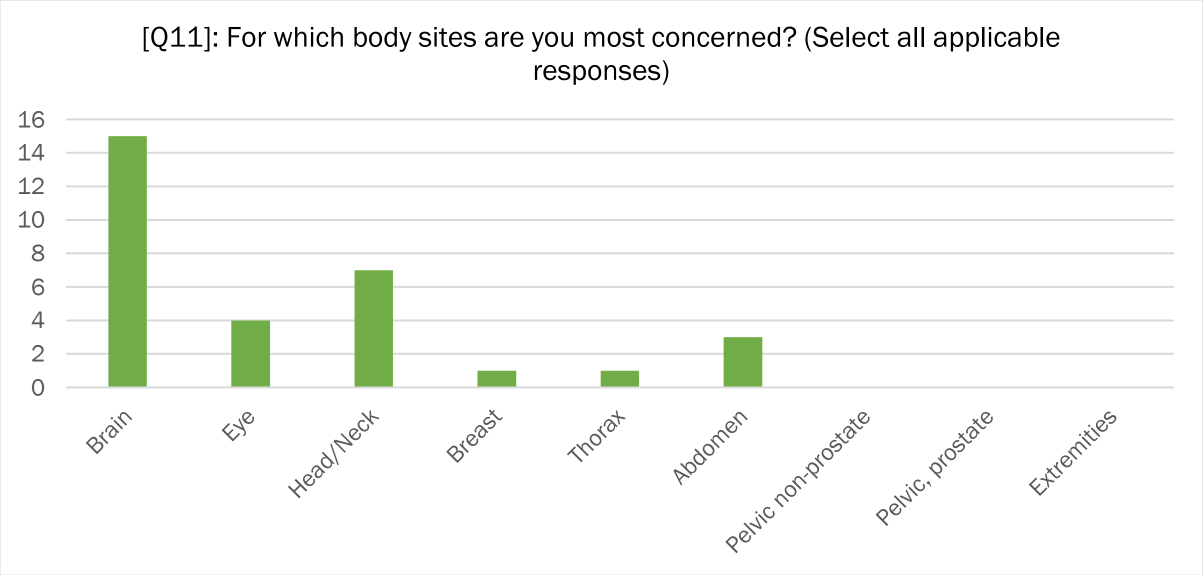
[Q11]: For which body sites are you most concerned? (Select all applicable responses)
0
2
4
6
8
10
12
14
16
Brain
Eye
Head/Neck
Breast
Thorax
Abdomen
Pelvic non-prostate
Pelvic, prostate
Extremities

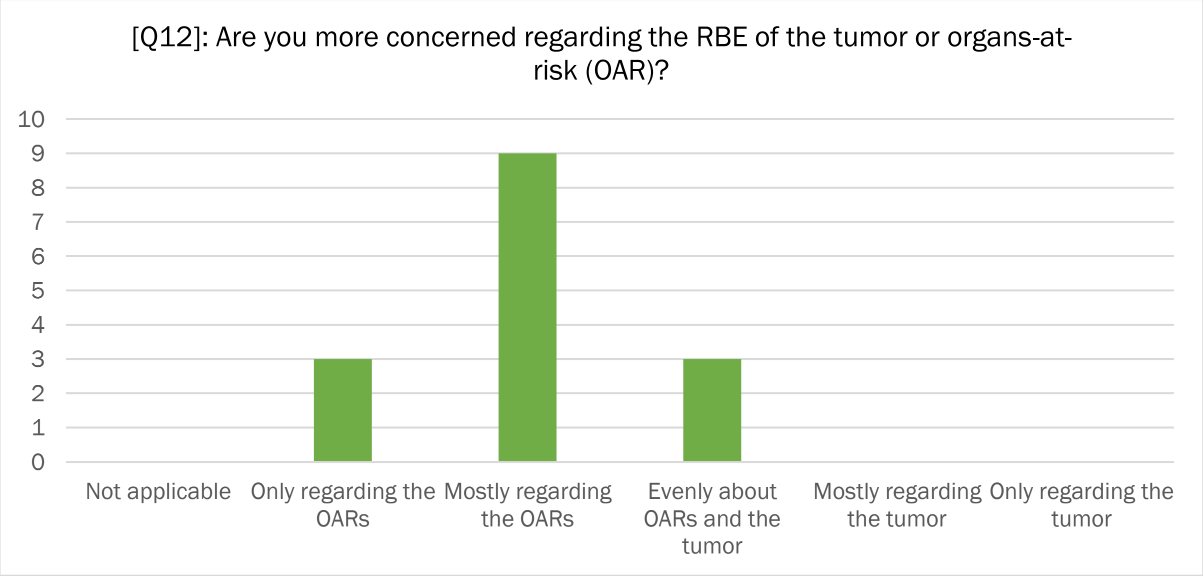
[Q12]: Are you more concerned regarding the RBE of the tumor or organs-at-risk (OAR)?
0
1
2
3
4
5
6
7
8
9
10
Not applicable
Only regarding the OARs
Mostly regarding the OARs
Evenly about OARs and the tumor
Mostly regarding the tumor
Only regarding the tumor

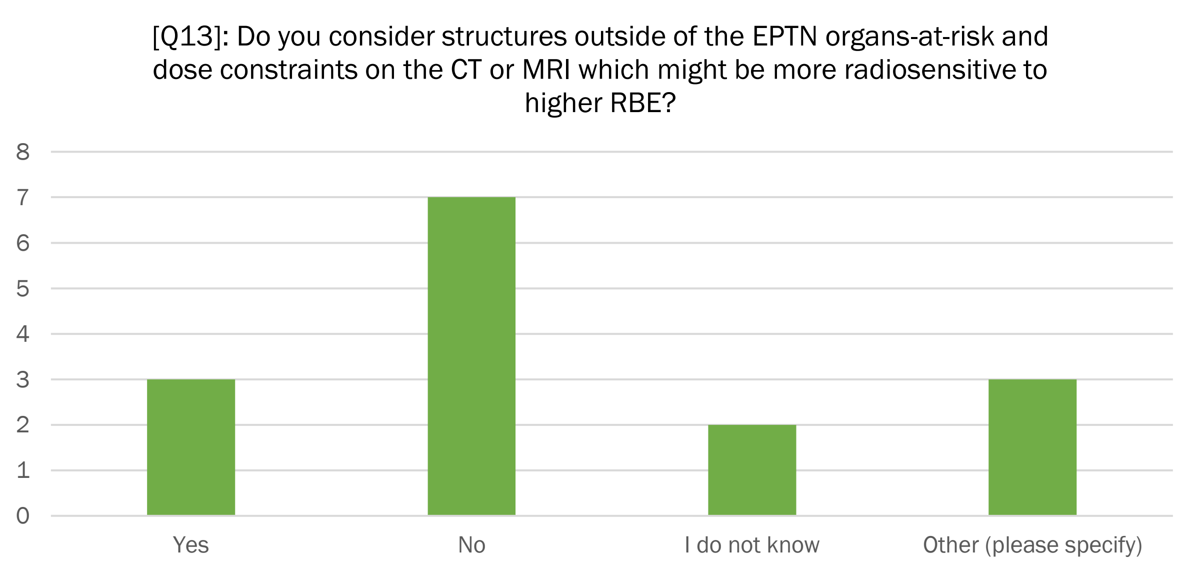

Specification of “Other (please specify)” answers:

- the periventricular space has been added to the EPTN OAR atlas but currently there is no reasonable recommendation for dose constraints available
- Not presently but neurophysiology and multimodality MRI informations including on memory and connectivities is constanrly evolving so we should keep eyes open
- Oral and intestinal, mucosa, cartilage, bone

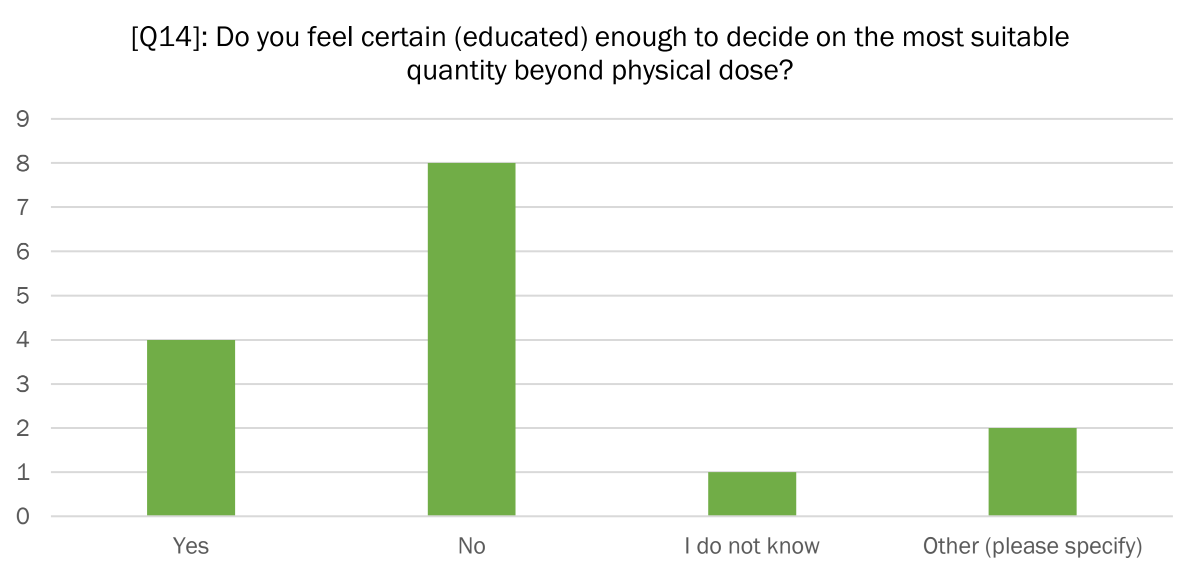

Specification of “Other (please specify)” answers:

- At least I feel that I could manage other quantities and their associated uncertainties
- LET achievable RBE more subject to compexity do to be discussed further with better knowledge

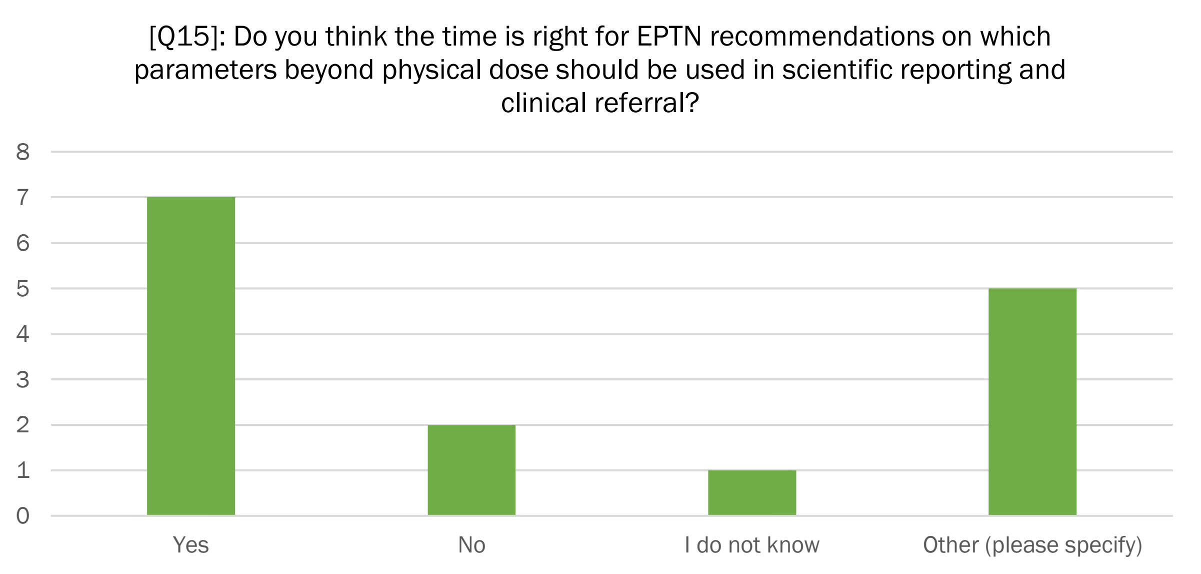

Specification of “Other (please specify)” answers:

- No for clinical practise, yes for scientific reporting
- Reporting is important but clinical outcomes are extremely important, too early for recommendations
- based on scientific evidence deside which parameters are relevant to report
- May more mature data base and interactions
- I think we need to better understand the clinical significant of adopting quantities beyond physical dose. We need to understand its relationship with the outcome (tumor control and tox)

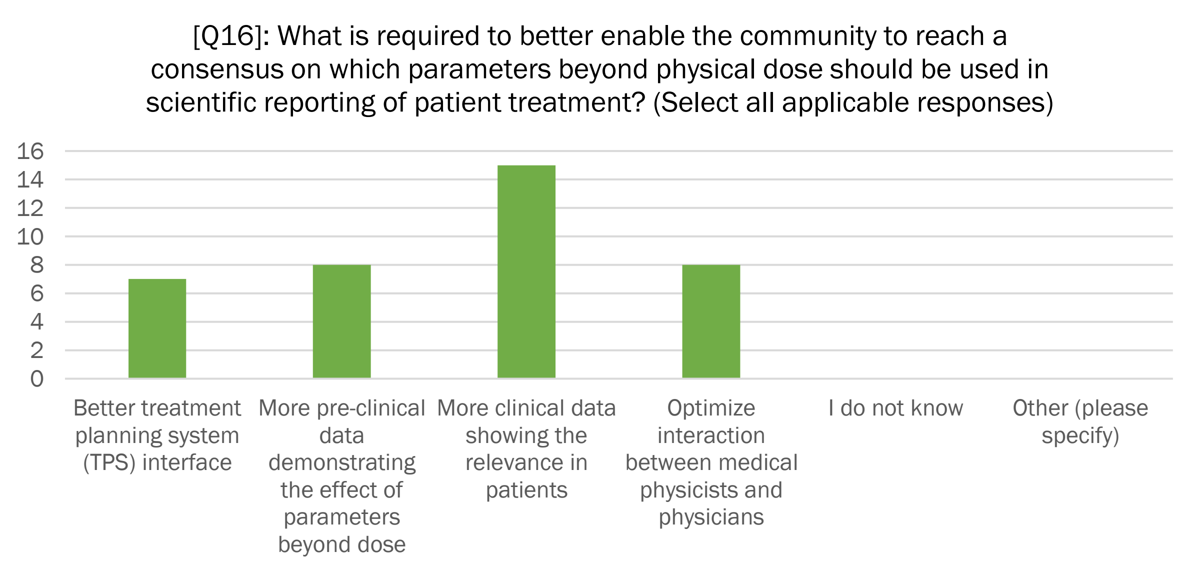

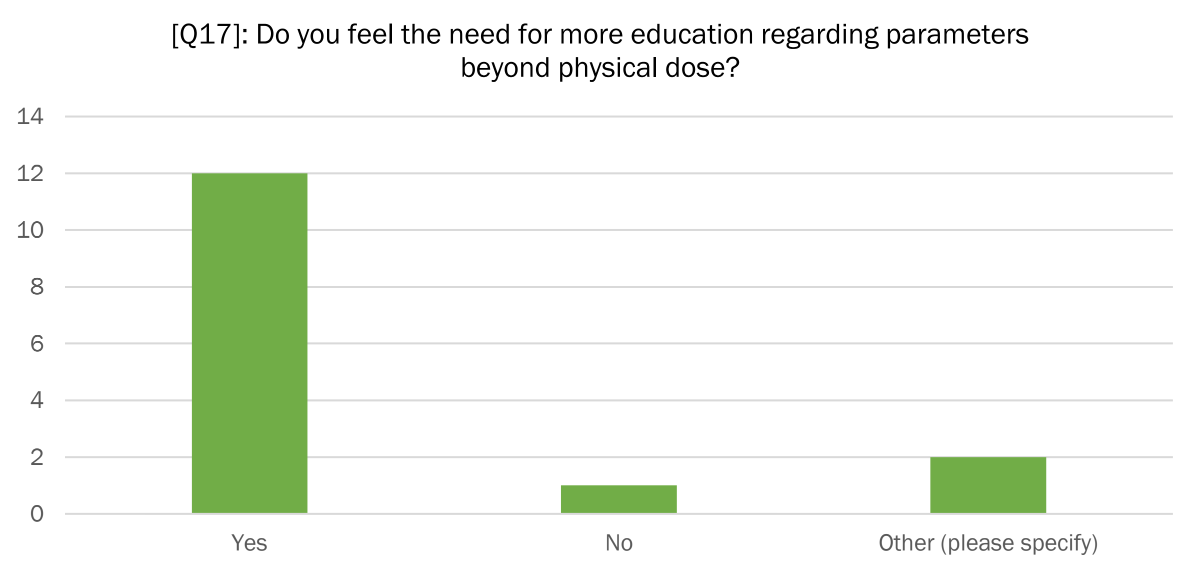

Specification of “Other (please specify)” answers:

- I would say yes but in our institution both clinicians and physicists are aware of the complexity of variable biological effectiveness given the experience coming from carbon ions and preparing helium ion irradiation
  [For the purposes of quantitative analysis, this answer was classifed as “Yes”]
- I am quite expert on the topic, but I think most colleagues would need more education
  [For the purposes of quantitative analysis, this answer was classifed as “Yes”]

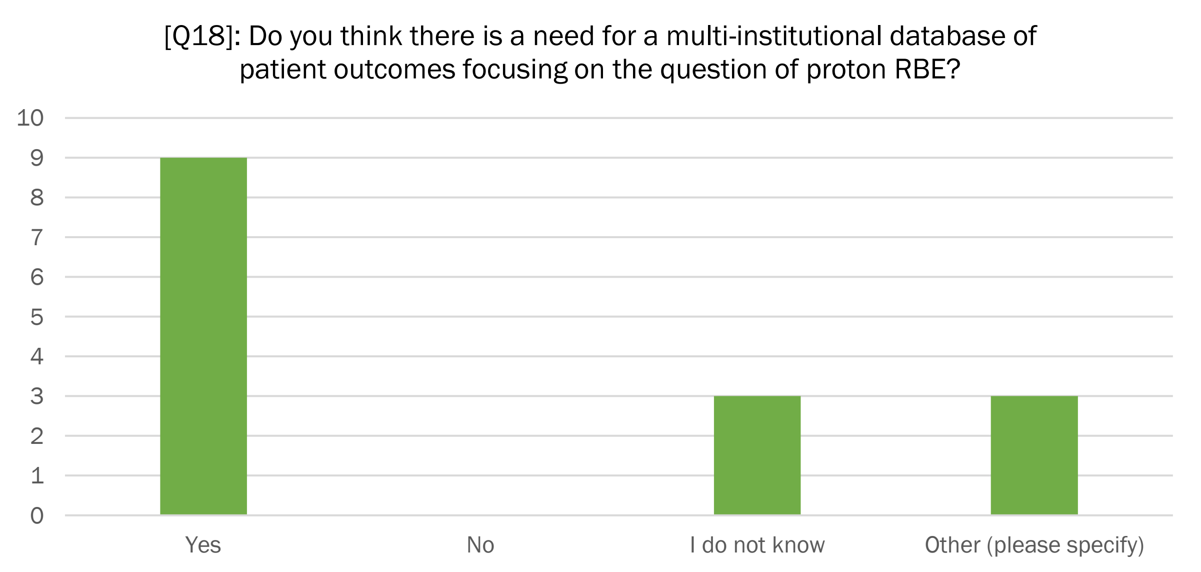

Specification of “Other (please specify)” answers:

- It is important to know what are the expectations of such multi-institutional data base, pros and cons and which questions will be answered based with the data base
- Sharing adverse toxicities outcome would be valuable but always a difficult issue
- Yes, but within a global multi-institutional database including these data
  [For the purposes of quantitative analysis, this answer was classifed as “Yes”]

Section 3: Physicist

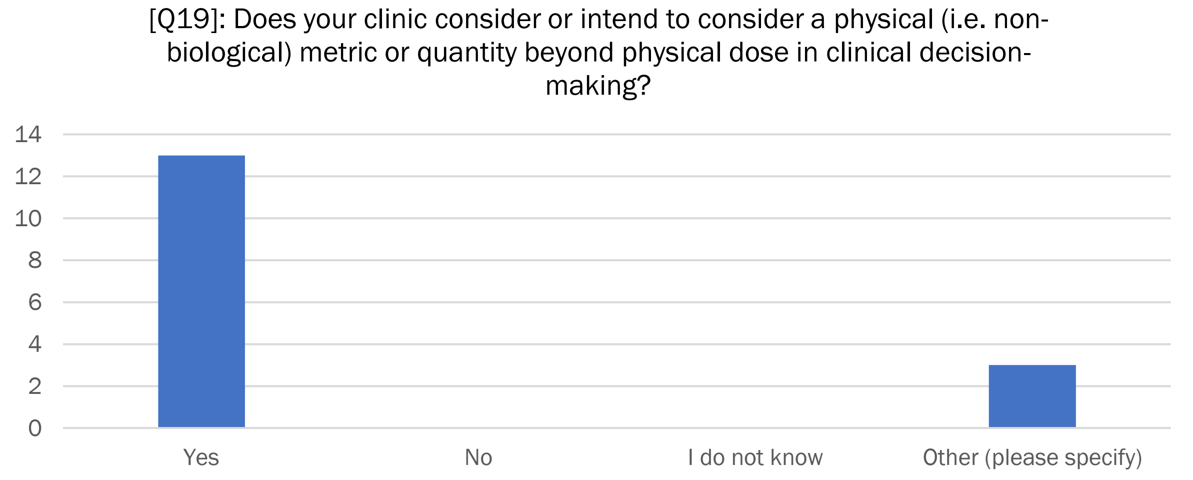

Specification of "Other (please specify)" answers:

- D*LETd and LETd are calculated, but hardly used
- At the time being no non-biologic metric is used in clinical practice, but we are testing LET optimization approaches on a TPS research version
- LET has been used for retrospective analyses and even prospectively in individual cases.
  [For the purposes of quantitative analysis, this answer was classifed as "Yes"]

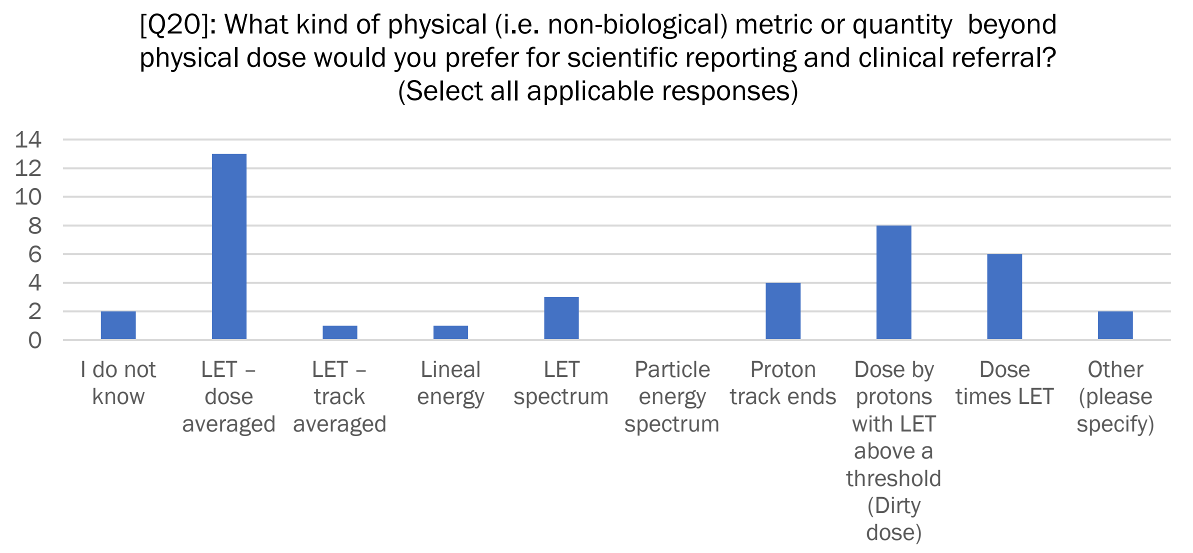

Specification of "Other (please specify)" answers:

- Variable RBE models
- In discussion, probably dirty dose
  [For the purposes of quantitative analysis, this answer was classifed as "Dose by protons with LET above a threshold (Dirty dose)"]

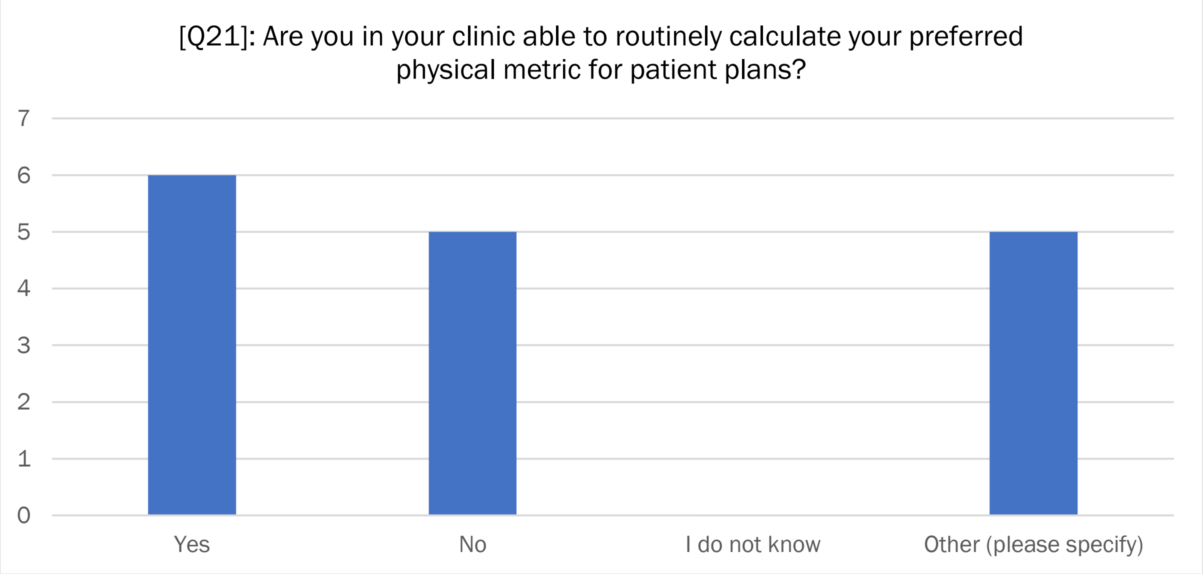

Specification of “Other (please specify)” answers:

- Only in research TPS or Monte Carlo
- For some metrics (LETd, d*LET yes, dirty dose not routinely)
- It is possible with an in-house developed Monte Carlo solution to calculate LETd, LET weighted dose and RBE models but not performed routinely
- In the road map, probably by end of 2024
- Not routinely, but in case of need we have commercial (TPS research version) and not commercial tool to score the dose average LET

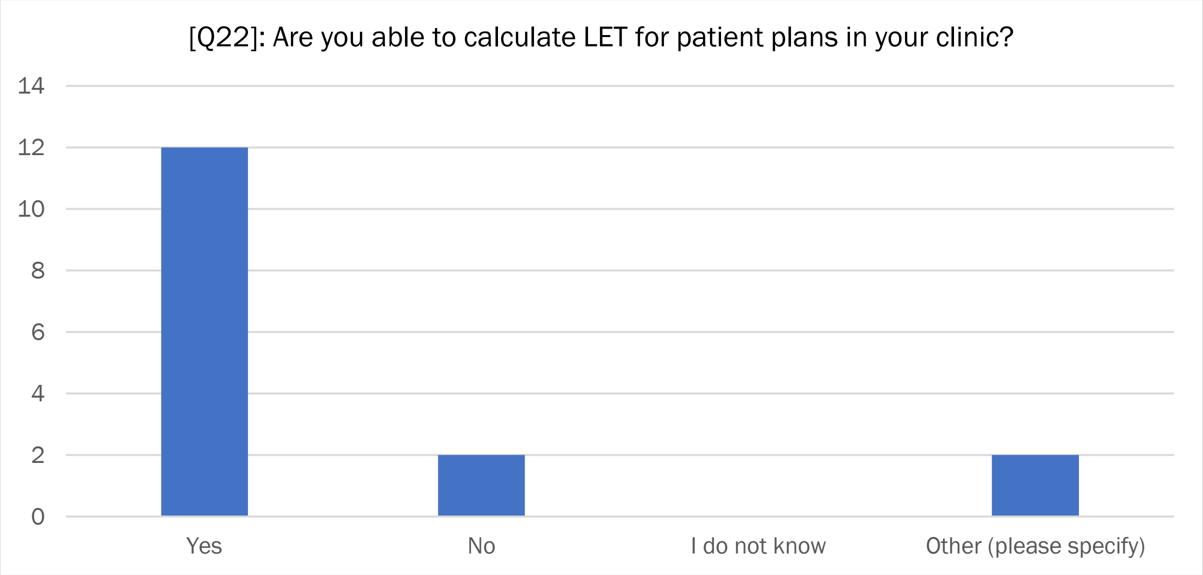

Specification of “Other (please specify)” answers:

- Yes, but not routinely
- In case of need we have commercial (TPS research version) and not commercial tool to score the dose average LET

[Q23]: What software package do you use for the calculation?

Specification of “Other (please specify)” answers:

- Raystation Research and TOPAS Monte Carlo
- MC2
- RayStation 10B in clinical routine, RayStation 9A-IonPG in research
- RayStation
- RayStation 11B
- RayStation proton MC, FLUKA MC (protons, C- and He-ions)
- RayStation, Fred (Monte Carlo package)
- RayStation and scripting
- In house developed Monte Carlo based on GATE (Geant4)
- Eclipse V15.6
- RaySearch Raystation TPS
- Raystation v12 research version
- RayStation, openMCSquare, psiplan
- RayStation

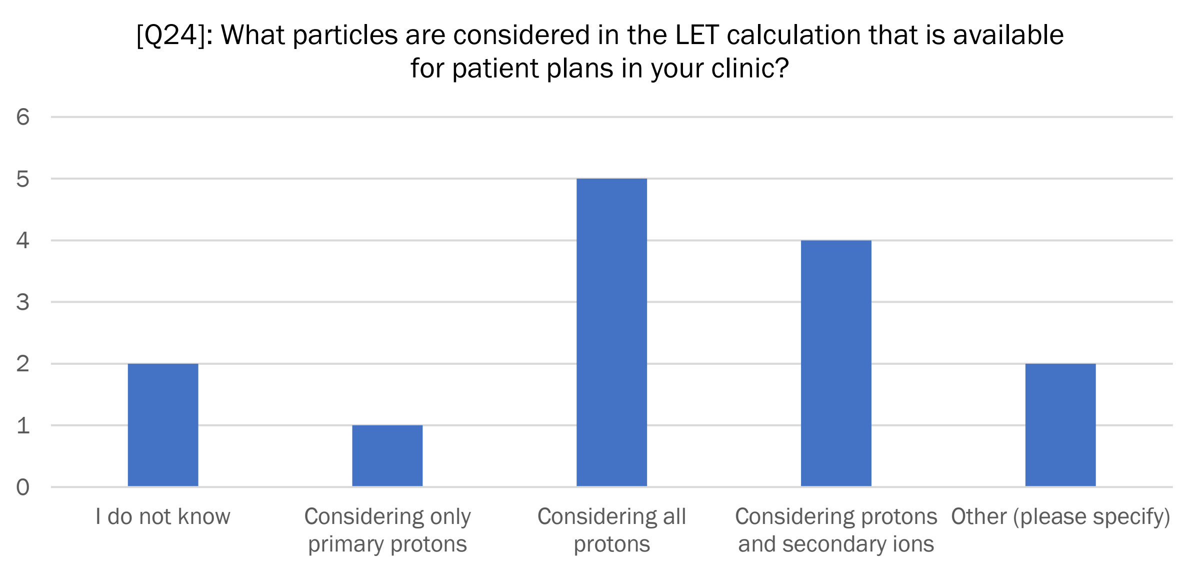

Specification of “Other (please specify)” answers:

- QGSP_BIC physics list
- should checked

[Q25]: What type of LET are you able to calculate for patient plans in your clinic? (Select all applicable responses)

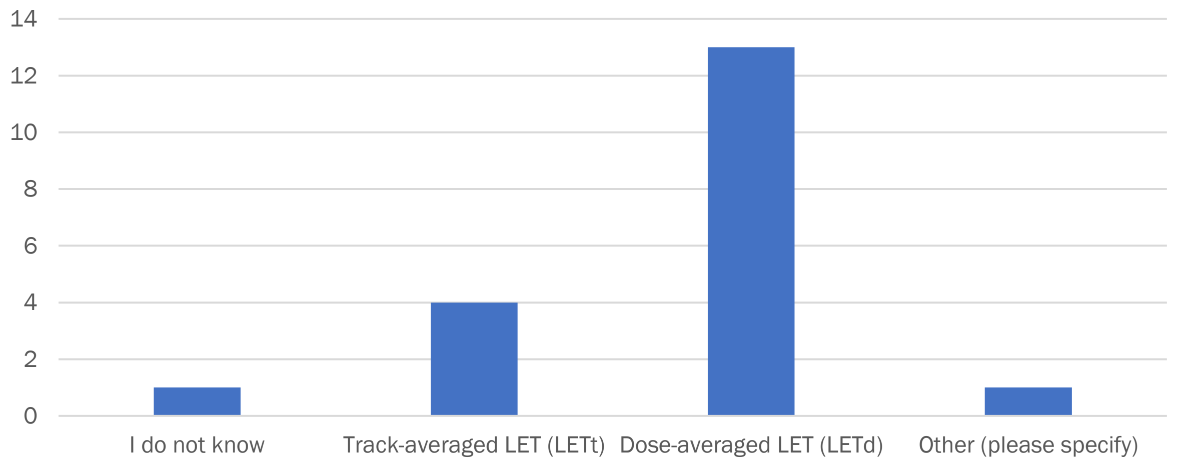

Specification of “Other (please specify)” answers:

- Dirty dose

[Q26]: How would you preferably communicate and receive the physical metric beyond physical dose?

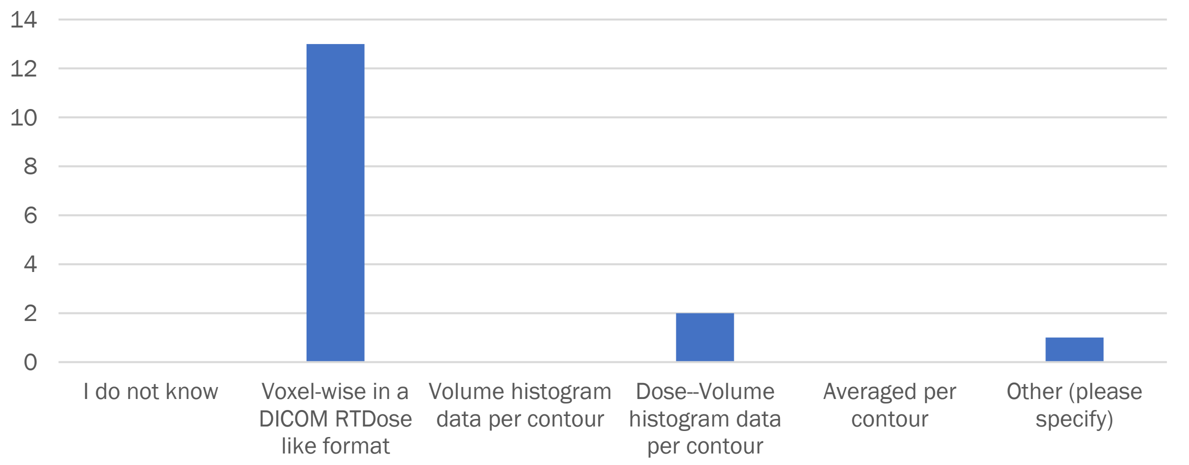

Specification of “Other (please specify)” answers:

- We should discuss internally. We didn't discuss the issue at such level of deteil

[Q27]: Do you think the time is right for EPTN recommendations on what physical (i.e. non-biological) metric beyond physical dose should be used in scientific reporting and clinical referral?

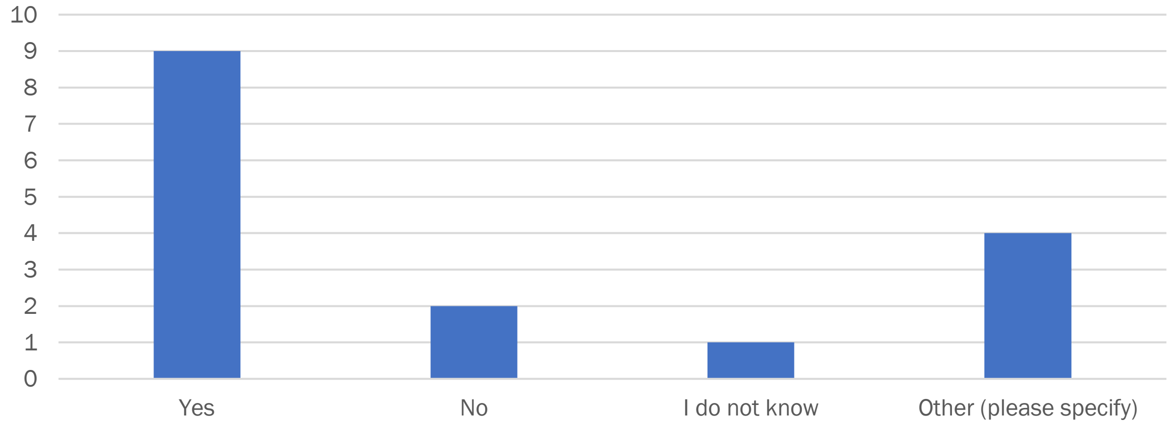

Specification of “Other (please specify)” answers:

- yes for scientific reporting
- Not yet, we need evidence-based data based on analysis of patient outcomes
- In general it could be useful, but the commercial solution should be at the same time otherwise the applicability in the clinical practice would remain far
- Clinical evidence is not yet mature.

[Q28]: Is your clinic able to calculate RBE using a variable RBE model for patient plans?

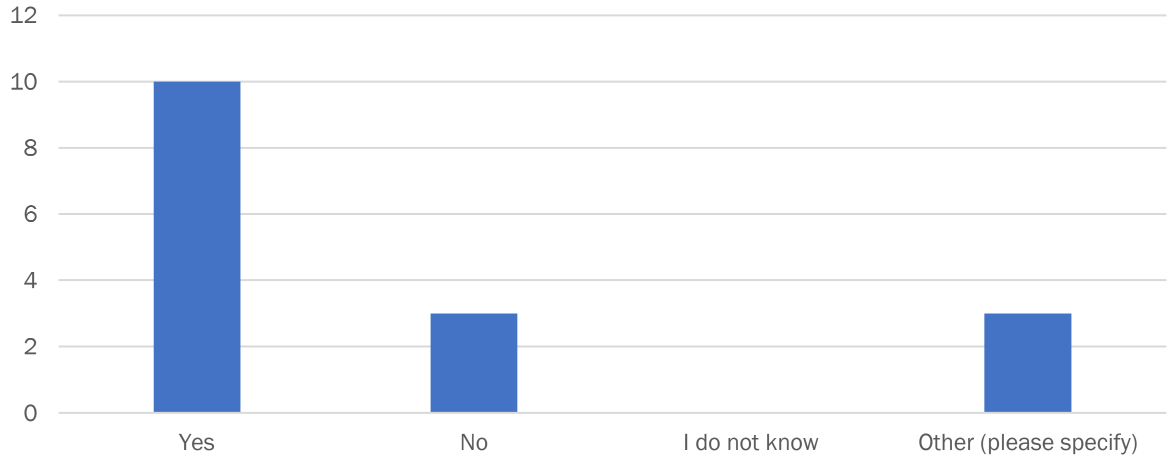

Specification of “Other (please specify)” answers:

- Yes, but not routinely
- Yes, but not routinely
- For research purposes.

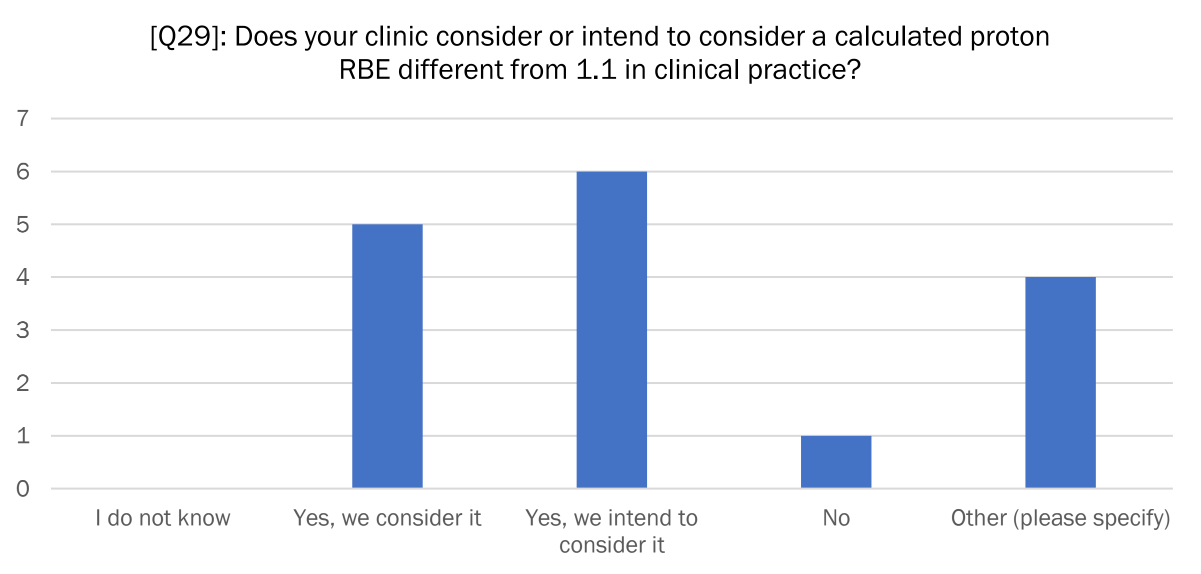

Specification of "Other (please specify)" answers:

- In principle yes, given enough evidence (currently still to weak)
- Not in clinical practise, but for plan evaluation
- In the mid-long term it could be an option, currently is not under discussion within the team
- A range of RBE values may be considered

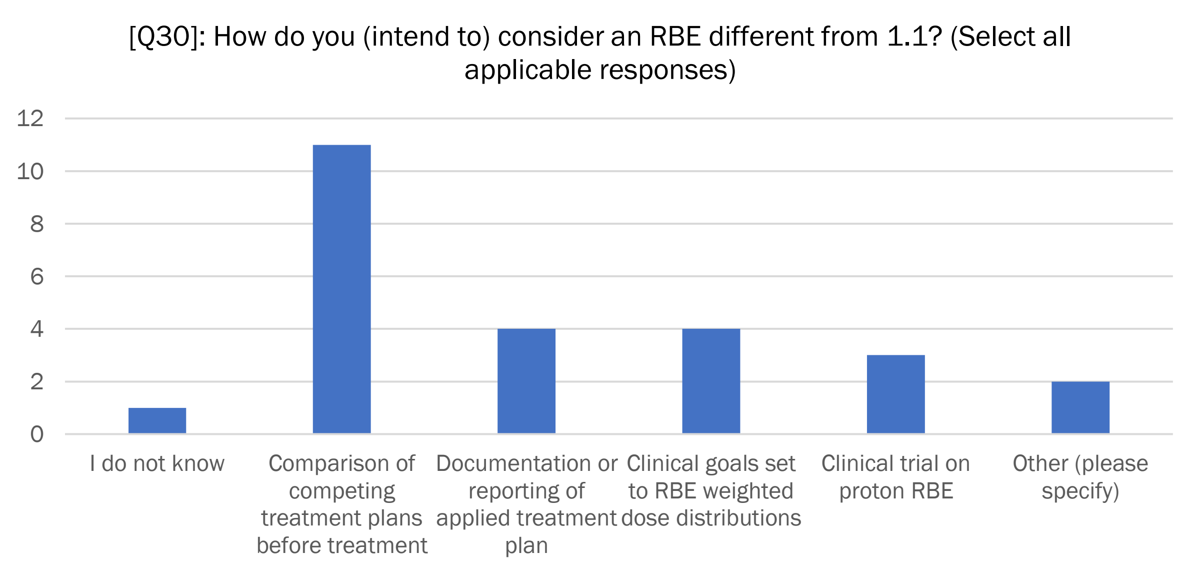

Specification of "Other (please specify)" answers:

- Retrospective analysis
- we mainly do this for carbon ions of course

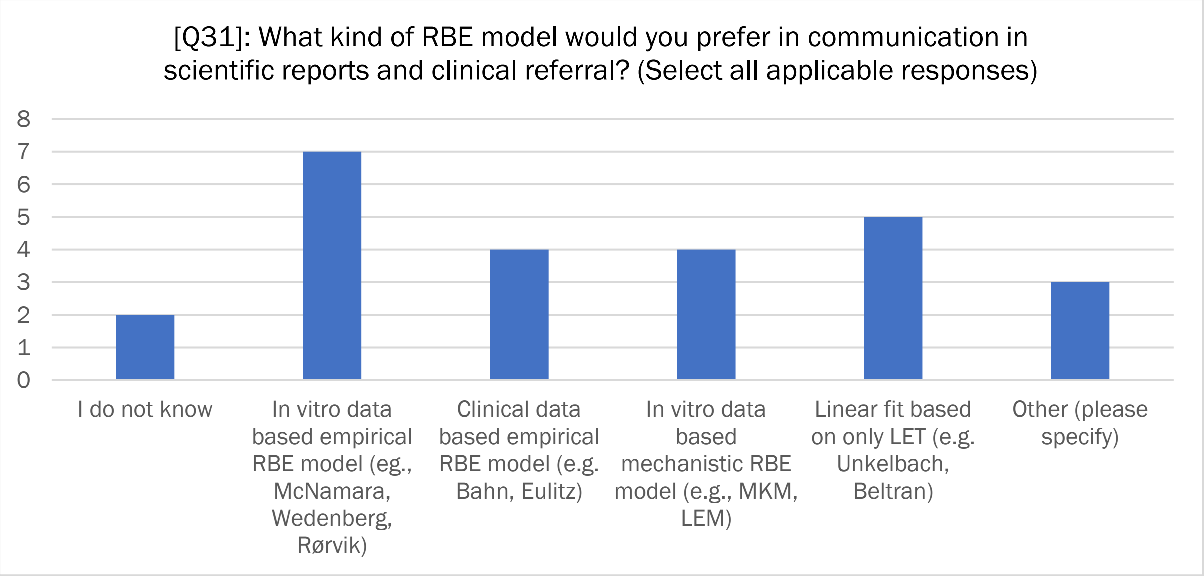

Specification of “Other (please specify)” answers:

- Externaly validated models based on clinical data
- ... clincal data based models where applicable, otherwise in-vitro in comparison
- Several models may be considered.

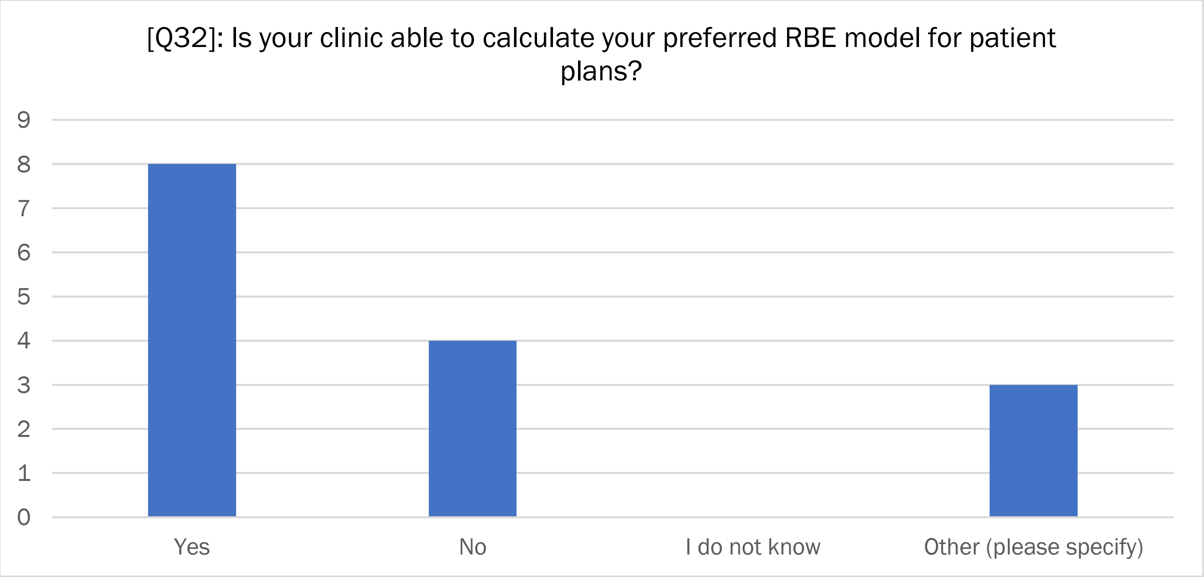

Specification of “Other (please specify)” answers:

- Yes, but not routinely.
- Not routinely
- For research purposes.

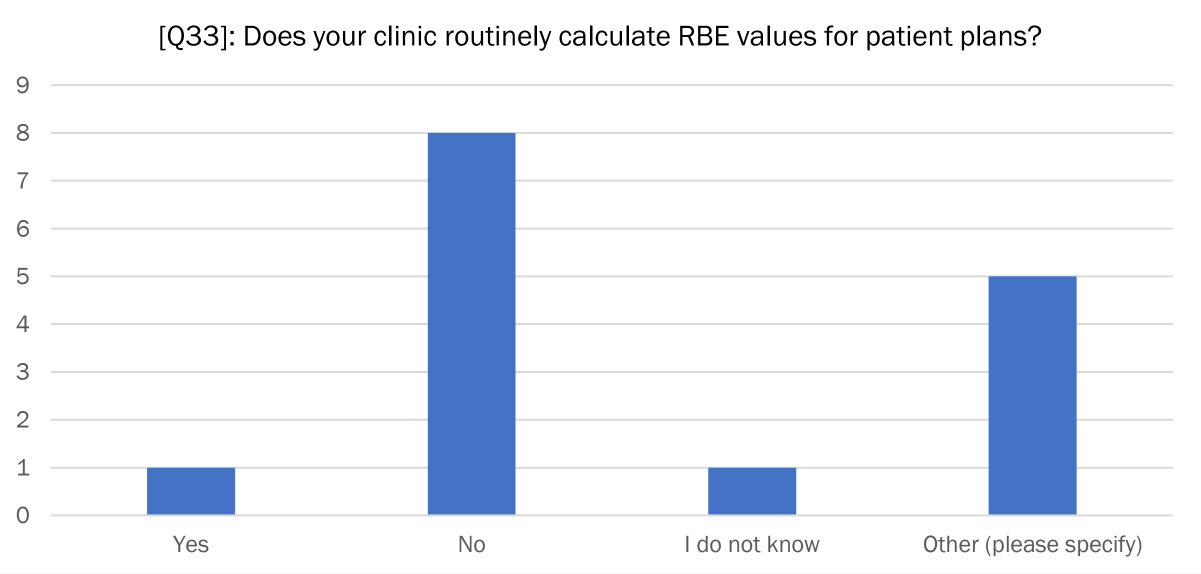

Specification of “Other (please specify)” answers:

- only selected cases
- in hypofractionation and reirradiation
- For challenging beam arranegements only (e.g. unilateral beams withserial OARs distal to the beams's path
- not clear the request
- mainly for carbon ions

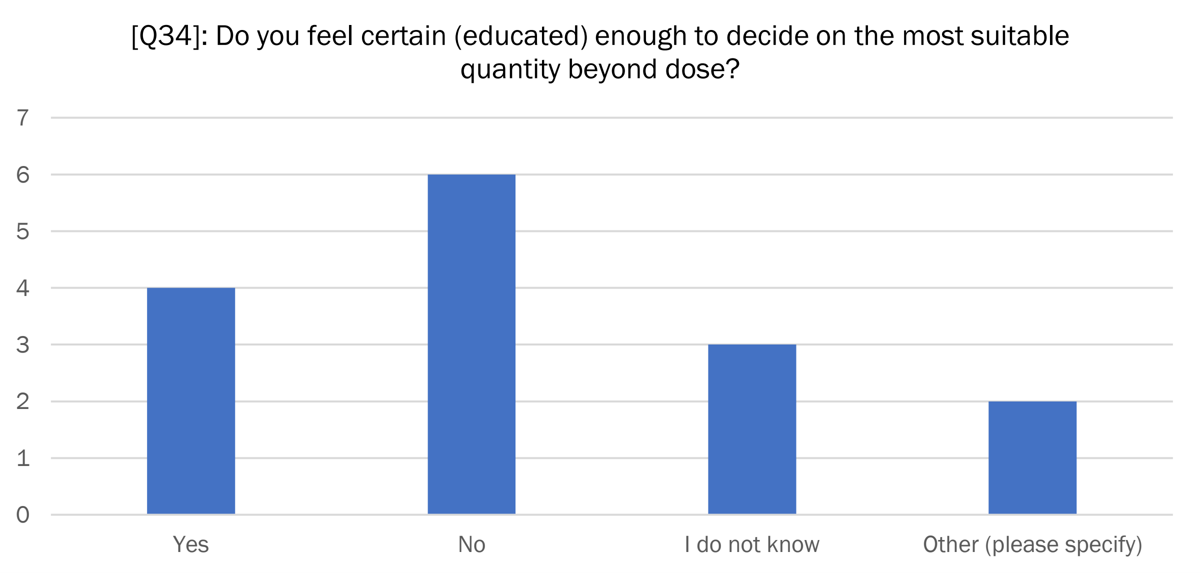

Specification of “Other (please specify)” answers:

- I think the statistical basis is lacking, but we also need to report parameters to build future knowledge
- I do believe that the discussion within our team is not enough mature

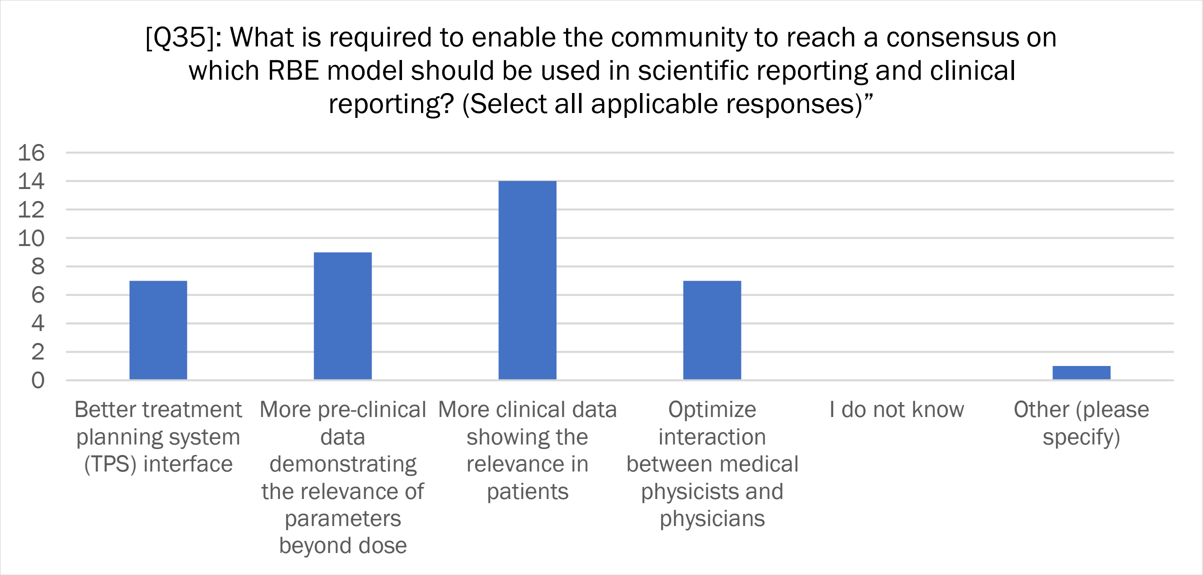

Specification of "Other (please specify)" answers:

- We should be able to reach such a consensus now

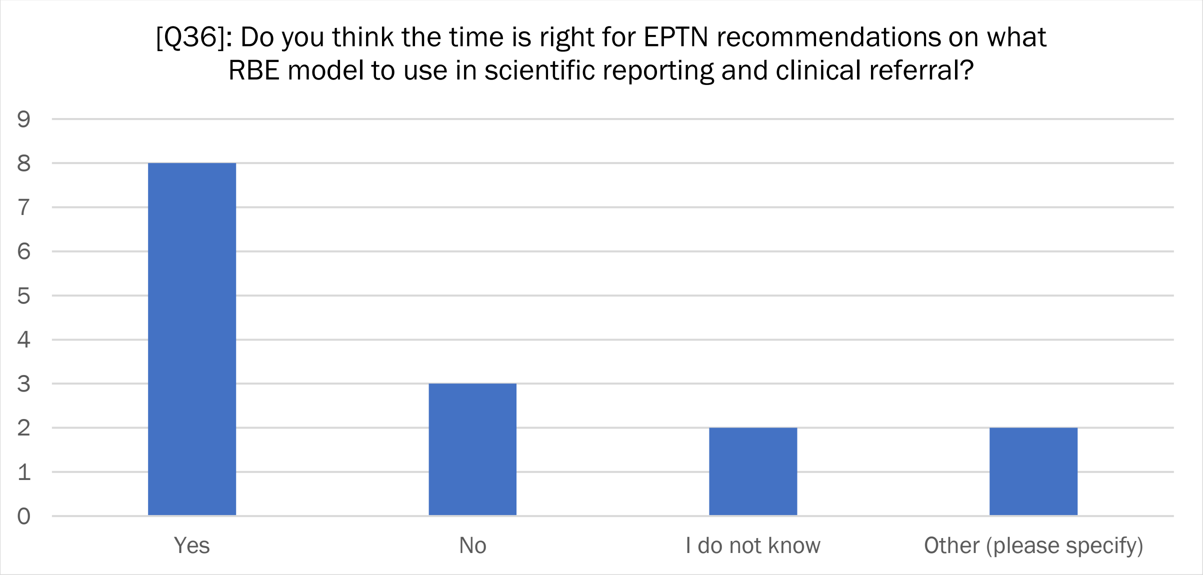

Specification of "Other (please specify)" answers:

- I think the time is right for scientific reporting, could be more than one level of reporting
- Probably yes, but this should come together with the availability of evaluation tools easily accessible and implementable in the clinical prctice

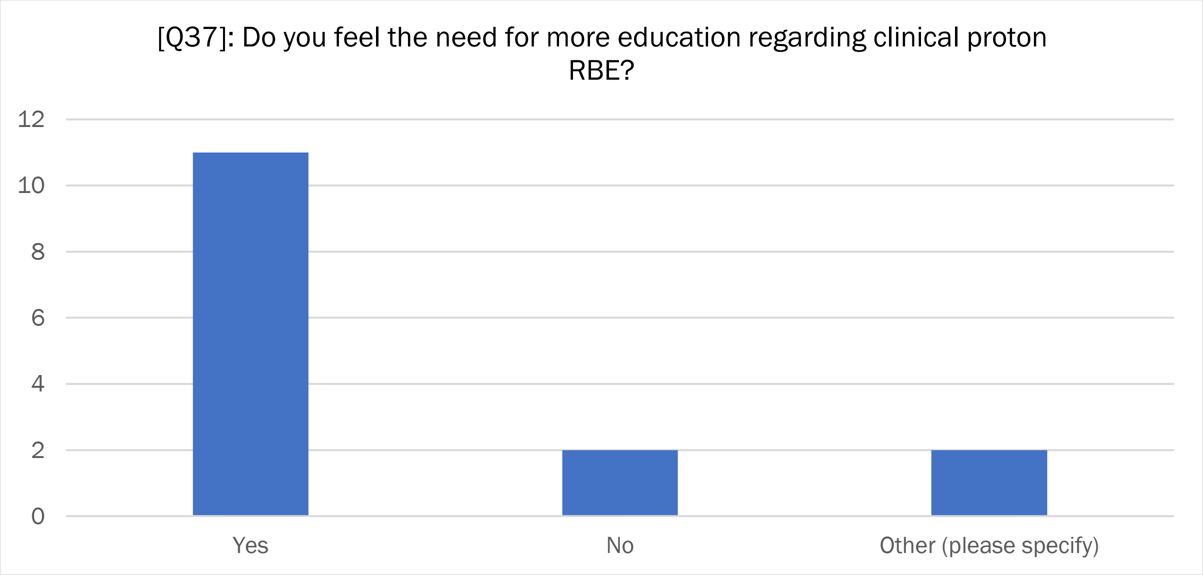

Specification of “Other (please specify)” answers:

- The community needs more knowledge of advanced statistical methods
- In general (for a broader MP audience, RTTs, RTOs) yes
  [For the purposes of quantitative analysis, this answer was classifed as “Yes”]

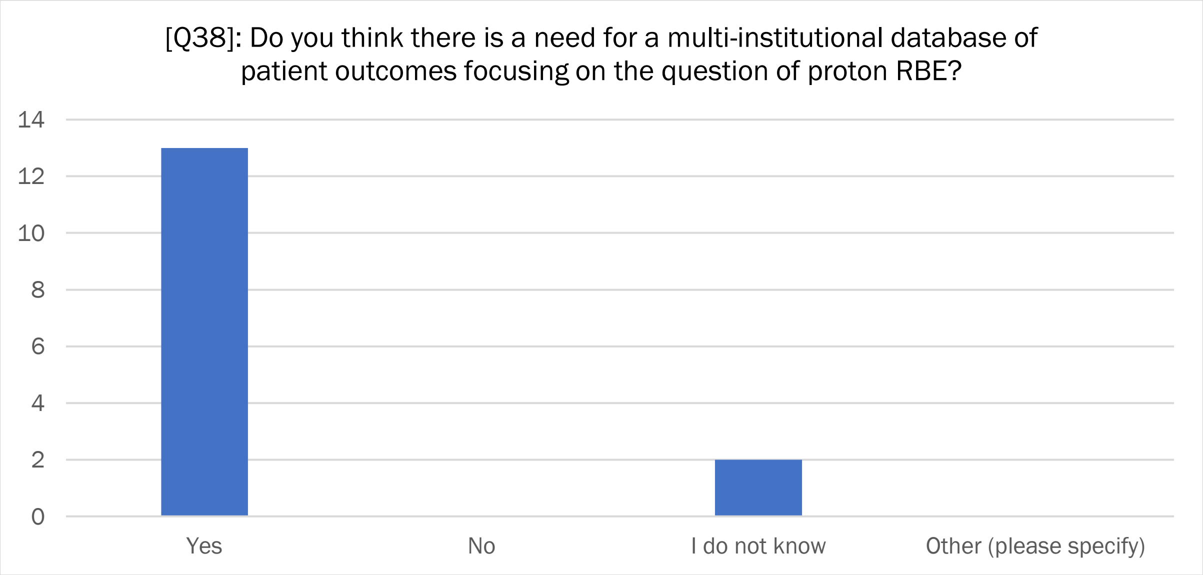

Supplement: Supplementary Data 1 [file mmc1.pdf]
